# Alterations of the Muscular Fatty Acid Composition and Serum Metabolome in Bama Xiang Mini-Pigs Exposed to Dietary Beta-Hydroxy Beta-Methyl Butyrate

**DOI:** 10.3390/ani11051190

**Published:** 2021-04-21

**Authors:** Changbing Zheng, Bo Song, Qiuping Guo, Jie Zheng, Fengna Li, Yehui Duan, Can Peng

**Affiliations:** 1 CAS Key Laboratory of Agro-Ecological Processes in Subtropical Region, Hunan Provincial Key Laboratory of Animal Nutritional Physiology and Metabolic Process, National Engineering Laboratory for Pollution Control and Waste Utilization in Livestock and Poultry Production, Institute of Subtropical Agriculture, Chinese Academy of Sciences, Changsha 410125, China; 20182025028@stu.scau.edu.cn (C.Z.); songbo20@mails.ucas.ac.cn (B.S.); guoqiuping15@mails.ucas.ac.cn (Q.G.); zhengjie202@mails.ucas.ac.cn (J.Z.); lifengna@isa.ac.cn (F.L.); 2 Guangdong Provincial Key Laboratory of Animal Nutrition Regulation, South China Agricultural University, Guangzhou 510642, China; 3 College of Advanced Agricultural Sciences, University of Chinese Academy of Sciences, Beijing 100039, China

**Keywords:** beta-hydroxy beta-methyl butyrate, intramuscular fat, n3 fatty acids, *N*-Methyl-l-glutamate, nummularine A, pigs

## Abstract

**Simple Summary:**

Pork is the most consumed meat source for humans, and the utilization of nutritional approaches to produce pork with an appropriate content of intramuscular fat (IMF) and a balanced ratio of different kinds of fatty acid is an important objective pursuit of swine production. We speculated that dietary supplementation of beta-hydroxy beta-methyl butyrate (HMB) may provide benefits in lipid metabolism of skeletal muscle. In this study, we try to investigate the effects of dietary HMB supplementation on muscular lipid metabolism in Bama Xiang mini-pigs. We found that HMB supplementation could decrease the IMF content and increase n3 polyunsaturated fatty acids as well as regulate the related metabolites (*N*-Methyl-l-glutamate and nummularine A) in the serum of Bama Xiang mini-pigs, thus improving their meat quality.

**Abstract:**

This study aimed to investigate the effects of dietary beta-hydroxy beta-methyl butyrate (HMB) supplementation on muscular lipid metabolism in Bama Xiang mini-pigs. Thirty-two piglets (8.58 ± 0.40 kg, barrow) were selected and fed a basal diet supplemented either with 0 (control), 0.13%, 0.64%, or 1.28% HMB for 60 days. Throughout the experiments, they had free access to clean drinking water and diets. Data of this study were analyzed by one-way ANOVA using the SAS 8.2 software package, followed by a Tukey’s studentized range test to explore treatment effects. The results showed that compared to the control, 0.13% HMB decreased the intramuscular fat (IMF) content and increased polyunsaturated fatty acids (PUFAs) in *Longissimus thoracis* muscle (LTM), and increased the n3 PUFAs in soleus muscles (SM, *p* < 0.05). Moreover, HMB supplementation led to alterations in the mRNA expression of genes related to lipid metabolism. Serum metabolome profiling showed that in both LTM and SM of Bama Xiang mini-pigs, *N*-Methyl-l-glutamate was positively correlated with SFA and nummularine A was negatively correlated with C18:3n3 PUFA (*p* < 0.05). Therefore, *N*-Methyl-l-glutamate and nummularine A might be potential biomarkers of the HMB-supplemented group. These results suggested that dietary HMB supplementation could decrease the IMF content and increase n3 PUFAs as well as regulate the related metabolites (*N*-Methyl-l-glutamate and nummularine A) in the serum of pigs.

## 1. Introduction

Pork is the most consumed meat source for humans, and hence there has been a growing focus on pork quality [1]. An increasing body of literature has identified intramuscular fat (IMF) and the fatty acid composition of muscle tissues as important contributing factors to pork quality [2,3,4,5]. There is a close relationship between IMF and meat edible qualities such as juiciness, flavor, drip loss, and tenderness [6]. The recommended range of IMF content is 2.5–3% [7]. Pork with low IMF content has poor palatability, whereas pork with high IMF content will greatly decrease the purchasing decisions of customers due to the existence of too rich visible marbling [8]. Meanwhile, the ratios of different kinds of fatty acids in muscle tissues are also central to the nutritional value of meat and contribute importantly to human health [9]. Specifically, excessive content of saturated fatty acid (SFA) and n6 polyunsaturated fatty acids (PUFA) together with increased ratios of SFA/PUFA and n6/n3 PUFA are associated with the development of cardiovascular and metabolic disorders in humans [10,11]. Nutritional modulation is an efficient strategy to alter the fatty acid profile and concentration of pigs’ muscles [3,12]. On this basis, the utilization of nutritional approaches to produce pork with an appropriate content of IMF and a balanced ratio of different kinds of fatty acid is an important objective pursuit of swine production.

Beta-hydroxy-beta-methyl butyrate (HMB), a metabolite of leucine, has gained popularity as a nutritional supplement in humans to augment the mass and strength of skeletal muscles [13] and in animals to modulate muscle fiber types as well as to improve carcass quality [14,15]. Apart from these roles, HMB also exerts important roles in the modulation of energy homeostasis and lipid metabolism in adipose tissues [16,17]. For example, in human studies, there is evidence that HMB led to a significant reduction in body fat (−1.1% vs. −0.5%) relative to a placebo, accompanied by a greater elevation in lean body mass (+1.4 kg vs. +0.9 kg) [18]. Similar results were obtained in high fat diet-fed mice, in which HMB supplementation (1% wt/vol) reduced high fat diet-induced body weight gain and the weight of white adipose tissues [17]. Further evidence comes from a swine model showing that HMB supplementation has the ability to modulate adipose tissue function such as lipolysis and fatty acid oxidation [16]. Of note, excessive leucine in tissues and blood may result in mitochondrial dysfunction and insulin resistance, thus increasing the future risk of developing diabetes and its related metabolic disorders. However, HMB cannot be converted reversibly to leucine and thus would not share similar functions to leucine in this regard [19,20]. Despite the importance of HMB in regulating lipid metabolism in adipose tissue, few studies have sought to investigate its effects on lipid metabolism in skeletal muscles of pigs. Therefore, it is posited that HMB may provide benefits in lipid metabolism of skeletal muscle similar to adipose tissue.

Metabolomics is an approach for analyzing quantitatively and qualitatively endogenous metabolites in a biological system; these metabolites are intermediates or the end products of cellular processes and have the ability to directly affect the phenotype [4,21]. How fatty acid composition of skeletal muscle is changed by dietary manipulations can be determined by a systematic study of metabolites and metabolic pathways.

Bama Xiang mini-pigs, an indigenous minipig breed from Bama Country of China, grow slowly and are lighter and fatter than the modern breeds such as Large White at slaughter, which makes them an ideal experimental animal model for biomedical research [22,23]. Notably, too-high fat content in skeletal muscle will greatly decrease the purchasing decisions of customers, thus decreasing the economic value of Bama Xiang mini-pigs [8]. Based on the roles of HMB in regulating lipid metabolism of adipose tissue, we hypothesized that HMB could improve lipid metabolism in skeletal muscle as well. Therefore, this study aimed to investigate the roles of dietary HMB supplementation in IMF, muscle fatty acid composition, the mRNA expression of lipid metabolism-related genes in skeletal muscle, and serum metabolomics in Bama Xiang mini-pigs.

## 2. Materials and Methods

### 2.1. Animals and Diets

All animal procedures were performed under the guidelines of the Committee on Animal Care of the Institute of Subtropical Agriculture, Chinese Academy of Sciences under ethic approval number ISA-2017-023.

Thirty-two Bama Xiang mini-pigs with similar initial body weight (barrow, 8.58 ± 0.40 kg; 60 ± 2 days) were chosen and randomly divided into four groups (with eight piglets per treatment), that is, basal diet (control group, CON); basal diet +0.13% HMB-Ca (low-dose, HMB1 group); basal diet +0.64% HMB-Ca (moderate-dose, HMB2 group); basal diet +1.28% HMB-Ca (high-dose, HMB3 group), in a completely randomized design according to the body weight. All diets were formulated to be isoenergetic and isonitrogenous and to meet the nutrient requirements and physiological needs of growing mini-pigs (Table 1) [24]. Pigs were housed individually in cages (0.8 × 1.8 m) throughout the experiment and had free access to clean drinking water and diets. The experiment lasted for 60 days.

### 2.2. Sample Collection

All the pigs were fasted overnight at the end of the feeding trial and slaughtered by electrically stunning (250 V, 0.5 A, for 5–6 s) and exsanguinating. Notably, before slaughter, serum samples were collected and then stored as previously described [2]. After slaughter, samples of the *Longissimus thoracis muscle* (LTM) and soleus muscle (SM) dissected from the left side of the carcasses were stored at −20 °C or placed in liquid nitrogen (about 10 g of each tissue) and then stored at −80 °C until further analysis.

### 2.3. Measurements of Serum Amino Acids and Lipid-Related Substances

Amino acid concentrations in serum were determined as previously described [25]. The concentrations of glucose-6-phosphate dehydrogenase (G6PD), adipose tissue triglyceride lipase (ATGL), lipoprotein lipase (LPL), and 3-hydroxy-3-methylglutaryl coenzyme A reductase (HMGR) were measured with the corresponding commercial ELISA kits (Jiangsu enzymebiao Biotechnology Co., Ltd., Jiangsu, China) according to their instructions by using an enzyme-linked immunosorbent assay plate reader (Bio-Tek, Winooski, VT, USA).

### 2.4. Intramuscular Fat and Fatty Acid Composition

The IMF content of LTM and SM was analyzed using the Soxhlet extraction method as previously described [2]. The fatty acid composition of selected muscle tissues was analyzed via gas–liquid chromatography of methyl esters using an Agilent 7890A GC as previously described [26]. The concentration of individual fatty acids was expressed as a percentage of total fatty acids. Based on the fatty acid composition, we calculated the following parameters: the sum of SFAs and PUFAs; the PUFAs/SFAs ratio; the n6/n3 PUFA ratio; hypocholesterolemic/hypercholesterolemic ratio (h/H); atherogenicity index (AI); and thrombogenicity index (TI).

### 2.5. Quantitative Real-Time PCR Analysis

The quantitative real-time PCR analysis was conducted in LTM and SM samples as previously described [27]. Briefly, the TRIzol reagent (Invitrogen, Carlsbad, CA, USA) was used to isolate the total RNA of LTM and SM. The RNA quality and quantity were checked by 1% agarose gel electrophoresis and by ultraviolet spectroscopy using a NanoDrop^®^ ND-1000 spectrophotometer (Thermo Fisher Scientific, Inc., Waltham, MA, USA). Thereafter, approximately 1.0 μg of total RNA was used to be reverse-transcribed to complementary DNA by using First-Strand cDNA Synthesis Kit (Fermentas Inc., Hanover, MD, USA). The primers for the target genes were purchased from Sangon Biotech Co., Ltd., (Shanghai China) and their sequences are shown in Table S1. The relative expression levels of the selected genes were analyzed using SYBR Green Detection Kit (TaKaRa). The reaction system used for PCR detection includes the following substances: 5 μL SYBR Green mix, an aliquot (2 μL) of a complementary DNA template (corresponding to 25 ng of total RNA) solution, 0.2 μL ROX Reference Dye (50×), and 0.2 μL each of forward and reverse primers. PCR conditions were as follows: incubation for 10 min at 95 °C, followed by 40 cycles of denaturation for 15 s at 95 °C, annealing and extension for 60 s at 56–64 °C. The fluorescent signal was detected by the ABI 7900HT RT-PCR system (Applied Biosystems, Branchburg, NJ, USA). A melting curve was generated for each sample at the end of each run to ensure the purity of the amplified products. The values of cycle threshold (C_t_) represent the means of triplicate measurements. The mRNA expression levels of target genes were normalized by the reference gene β-actin (an internal control) and determined by the 2^−ΔΔCt^ method [27].

### 2.6. Liquid Chromatography—Tandem Mass Spectrometry (LC-MS/MS) Analysis

Serum samples (100 μL) of the CON and 0.13% HMB groups (*n* = 6) were spiked with 20 μL of internal standards (0.3 mg/mL L-2-chlorophenylalanine in acetonitrile) and 400 μL of acetonitrile:methanol (1:1, *v*/*v*) solution, and then extracted according to the manufacturer’s instructions (Majorbio Bio-Pharm Technology Co., Ltd., Shanghai, China). LC-MS/MS analysis for the extracted samples was performed using a Vanquish UHPLC system (Thermo Fisher) coupled with an Orbitrap Q Exactive HF-X mass spectrometer (Thermo Fisher Scientific, Inc.) operating in the data-dependent acquisition mode. The quality control (QC) sample was a pooled sample in which aliquots of all the extracted samples were mixed, and then analyzed using the same method as used for the analytic samples. It helped to represent the whole sample set, which would be injected at regular intervals (every 4 samples) in order to monitor the stability of the analysis. As presented in Figure S1A,B, the total ion chromatogram of the QC samples in positive and negative ion modes showed a good overlap in both ion modes, suggesting good stability of the proposed method.

The parameters of chromatography were as follows: column: BEH C18 (100 mm × 2.1 mm i.d., 1.7 μm; Waters, Milford, MA, USA); gradient mobile phase: water with 0.1% formic acid as solvent A and acetonitrile and isopropanol (1:1, *v*/*v*) (containing 0.1% formic acid) as solvent B; flow rate: 0.4 mL/min; sample injection volume: 2 μL; column temperature: 40 °C. The gradient mobile phase program applied as follows: t = 0 min, 95% A; t = 3 min, 80% A; t = 9 min, 5% A; t = 13.1 min, 95% A; t = 16 min, 95% A. The parameters of the mass spectrum were as follows: sheath gas flow rate 40 psi; aus gas flow rate 10 psi; aus gas heater temperature, 400 °C; ion-spray voltage floating (ISVF), −2800 V in negative mode and 3500 V in positive mode, respectively; normalized collision energy, 20–40–60 V rolling for MS/MS. The detection was carried out over a mass range of 70–1050 m/z.

### 2.7. Statistical Analysis

For the metabonomics data, the raw data were imported into the Progenesis QI software (Waters Corporation, Milford, MA, USA) for data preprocessing after UHPLC-QE-HFX/MS analyses. In order to detect metabolites, we carried on the principal component analysis (PCA) and orthogonal partial least-squares (OPLS-DA) analysis. To reveal the differences in the metabolic composition among groups, T-test (Student’s test) and the multivariate analysis of OPLS-DA were used to explore the potential marker or differential metabolites (variable importance in the projection (VIP) > 1, *p*-value < 0.05). For analysis software, the online platform of Majorbio ISanger Cloud platform (https://cloud.majorbio.com/) was used (assessed on 6 February 2020).

Data of this study were analyzed by one-way ANOVA using the SAS 8.2 software package, followed by a Tukey’s studentized range test to explore treatment effects. Results are presented as means ± standard errors (SD). Differences between significant means were viewed to be statistically different at *p* < 0.05.

## 3. Results

### 3.1. Growth Performance and IMF

All groups of pigs exhibited similar average daily feed intake and feed conversion ratios (*p* > 0.05, Table S2, https://kns.cnki.net/kcms/detail/11.5461.S.20210316.0920.012.html, assessed on 30 October 2020). However, compared to the CON, 0.13% HMB significantly increased the average daily gain (ADG) (*p* < 0.05). When fed to pigs at dosages of 0.64% and 1.28%, HMB failed to elevate ADG of pigs (*p* > 0.05, Table S2). The IMF content of the LTM in the 0.13% HMB group was significantly lower than that in the other three groups (*p* < 0.05), and no differences were observed among the three groups (*p* > 0.05, Table S2).

### 3.2. Serum Amino Acid Profile and Lipid-Related Substances

As shown in Table 2, relative to the CON, the 0.13% HMB augmented the serum concentrations of alanine, O-phosphoserine, and ornithine (*p* < 0.05), decreased the concentrations of aspartic acid and β-alanine (*p* < 0.05), and tended to increase the concentrations of threonine (*p* = 0.0841) and total NEAA (*p* = 0.0867). Compared to the CON, HMB at the level of 0.64% significantly increased the concentration of histidine (*p* < 0.05). Compared to the CON, HMB at the levels of 1.28% significantly increased the concentrations of alanine and arginine (*p* < 0.05). Dietary treatments exerted no significant effects on the concentrations of other amino acids (*p* > 0.05).

As shown in Table 3, compared to the CON, the 0.13% and 1.28% HMB reduced the concentrations of G6PD and LPL (*p* < 0.05), while the 0.64% HMB increased the LPL concentration (*p* < 0.05). The ATGL concentration was the lowest in the 0.64% HMB group and highest in the 0.13% HMB group, with intermediate values in the CON and 1.28% HMB groups (*p* < 0.05), and no differences were detected between CON and 0.13% HMB groups. The HMGR concentration in the CON group was similar to that in the 0.13% HMB group (*p* > 0.05), and was significantly lower than that in 0.64% and 1.28% HMB groups (*p* < 0.05).

### 3.3. Fatty Acid Composition of Skeletal Muscle Tissue

As depicted in Table 4, dietary HMB supplementation greatly affected the fatty acid composition in the LTM. The concentrations of n6 PUFAs, such as C18:2 n6c, C20:3 n6, and C20:4 n6, were increased in all samples within the 0.13% HMB group compared with the CON (*p* < 0.01). Compared with the CON, C18:3 n3 and the sum of SFA were significantly increased and decreased in the 0.13% group (*p* < 0.01), respectively. The sum of PUFA, the ratio of PUFA to SFA, the sum of n3 PUFA and n6 PUFA contents, and the n6:n3 PUFA ratio in the 0.13% group were higher than those in the CON group (*p* < 0.01). Compared with the CON, the 0.13% HMB group significantly increased the h/H and decreased the AI and TI (*p* < 0.05).

The results regarding the fatty acid composition of the SM of pigs fed diets with different dietary HMB levels are presented in Table 5. No difference was observed in the contents of C18:2 n6c, C20:3 n6 and C20:4 n6 as well as the sum of n6 PUFA among the groups (*p* > 0.05). The C18:3 n3 content and the sum of n3 PUFA were the highest in the 0.13% HMB group and lowest in the 1.28% HMB group, with intermediate values in the CON and 0.64% HMB groups. The sum of SFA in the 0.13% HMB group was significantly lower than that in the other three groups (*p* < 0.01). The n6:n3 PUFA ratio was the highest in the CON and lowest in the 0.13% HMB group, with intermediate values in the other two groups (*p* < 0.05). Compared with the CON, the 0.13% HMB group significantly decreased the TI (*p* < 0.05). Unlike in the LTM, no significant difference was observed in PUFA contents, the PUFA:SFA ratio, the h/H, and AI in the SM among the groups (*p* > 0.05).

### 3.4. mRNA Expression of Lipid Metabolism-Related Genes in Skeletal Muscles

**Figure 1:**
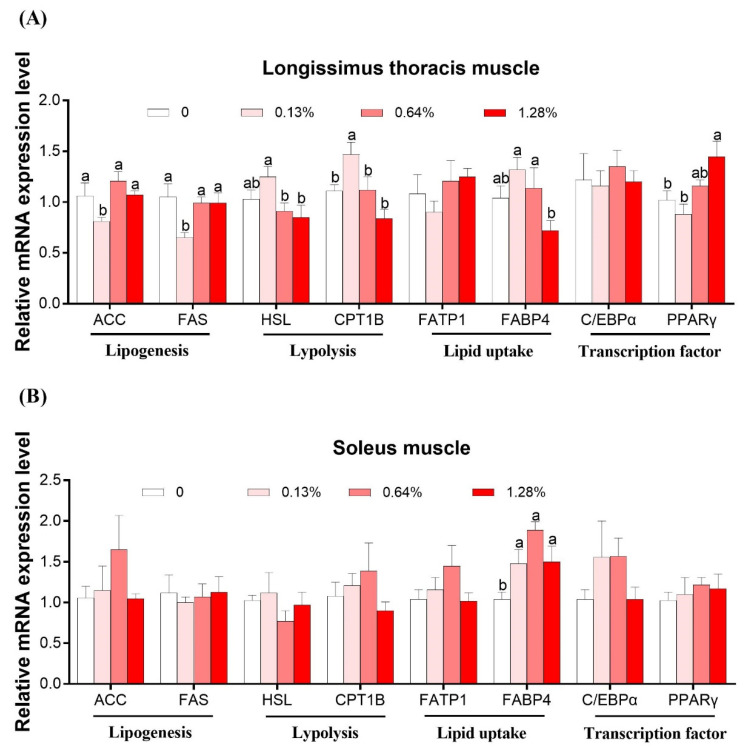
Effects of dietary HMB supplementation on the relative mRNA expression levels of genes in the *Longissimus thoracis muscle* (**A**) and *soleus* muscle (**B**) of the Bama Xiang mini-pigs (*n* = 8). The real-time PCR method was employed, and β-actin was used as an internal control. Values are means, with their standard errors represented by vertical bars. ^a,b^,Values within a row with different superscripts differ significantly (*p* < 0.05).

Figure 1 shows the mRNA expression levels of genes associated with lipogenesis (acetyl-CoA carboxylase, *ACC*; fatty acid synthase, *FAS*), lipolysis (hormone-sensitive lipase, *HSL*; carnitine palmitoyl transferase 1B, *CPT-1B*), lipid uptake (fatty acid transport protein, *FATP-1*; fatty acid-binding protein 4, *FABP4*), and transcription factor (peroxisome proliferator-activated receptor γ, *PPARγ*; CCAAT/enhancer-binding protein α, *C/EBPα*) in the LTM (Figure 1A) and SM (Figure 1B), respectively.

In the LTM, no difference was obtained in mRNA levels of *C/EBPα* among the groups (*p* > 0.05). Compared to the other three groups, the 0.13% HMB group increased the mRNA expression levels of *FAS* and *ACC*, and decreased those of *HSL* and *CPT-1B* (*p* < 0.05), and no difference was observed among the three groups. The *FABP4* mRNA expression level was the highest in the 0.13% and 0.64% HMB groups and lowest in the 1.28% HMB group, with an intermediate value in the CON group (*p* < 0.05). The *PPARγ* mRNA expression level was the highest in the 1.28% HMB group, followed by the 0.64% HMB group, and lowest in the other two groups (*p* < 0.05).

In the SM, the *FABP4* mRNA expression was increased in all the treatment groups relative to the CON (*p* < 0.05). No observable differences in the mRNA expression of *ACC*, *FAS*, *HSL*, *CPT1B*, *FATP1*, *C/EBPα*, and *PPARγ* were observed upon HMB supplementation (*p* > 0.05).

### 3.5. Metabolomics Analysis

**Figure 2:**
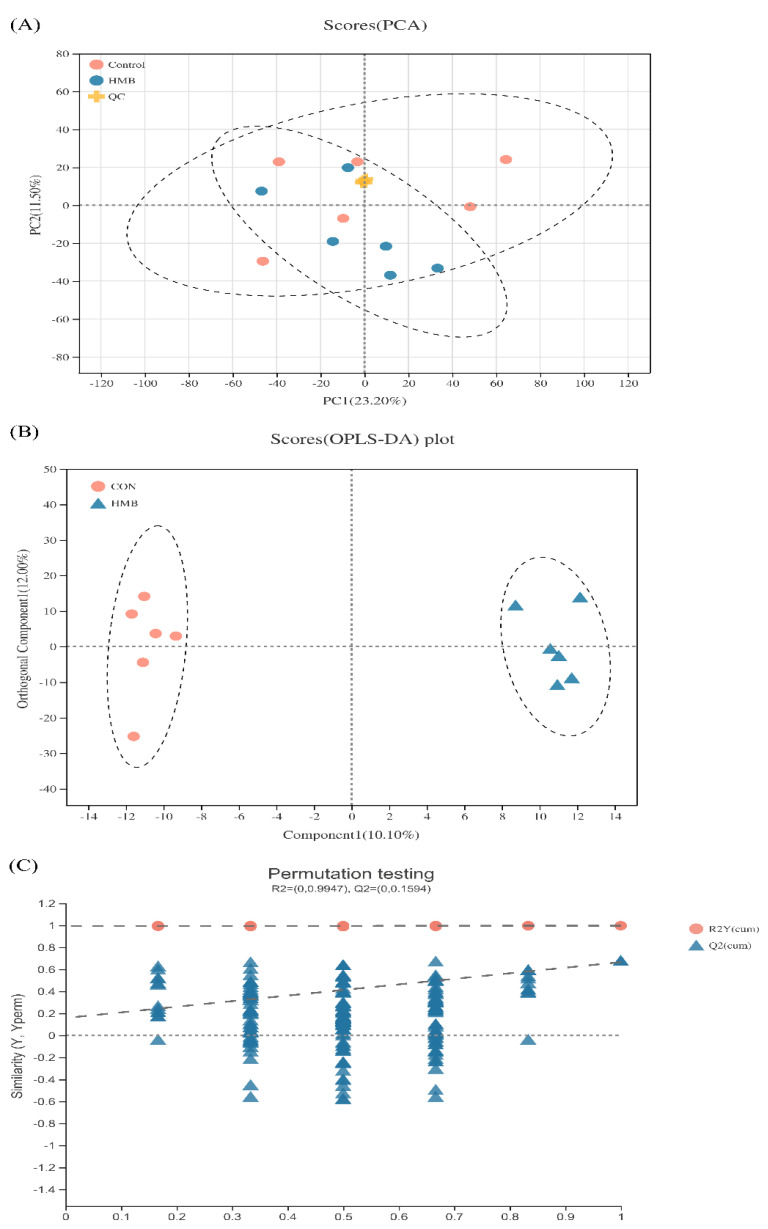
Principal component analysis (PCA) and orthogonal partial least-squares (OPLS-DA) score plots for serum metabolomics data (*n* = 6). (**A**) Principal component analysis (PCA) score plots based on serum metabolite profiles. Variances captured by the first and second principal components (PC) are shown. PC1 = 23.20%, PC2 = 11.50%. (**B**) Orthogonal correction partial least squares discriminant analysis (OPLS-DA) score plots for serum metabolite profiles. Through orthogonal rotation, OPLS-DA score graph can filter out the irrelevant information, so as to better distinguish the differences between groups and improve the performance of the model. Compent1 is the first prediction principal component decomposition degree, orthogonal Component1 is the first orthogonal decomposition degree. (**C**) OPLS-DA model validation diagram. The abscissa represents the replacement retention of the replacement test (the proportion consistent with the order of Y variables of the original model, the point with the replacement retention of 1 is the R2 and Q2 values of the original model), the ordinate represents the values of R2 (red dot) and Q2 (blue triangle) replacement test, and the two dashed lines show the regression lines of R2 and Q2, respectively.

To further determine the slight differences in metabolic profiles among all groups, PCA and OPLS-DA were carried out. As shown in Figure 2A, the scatter plots of serum were cross-distributed among the two groups. Even so, we cannot draw conclusions that no differences were observed in the metabolic profiles of serum among the groups. Supervised OPLS-DA can reveal the specific variables that caused differences among groups relative to unsupervised PCA [28]. Therefore, the multivariate statistical analysis using OPLS-DA was further performed to detect serum-specific metabolites that differ between the CON and HMB treatment, and the scatter plots are presented in Figure 2B. The scatter plots of the CON and HMB groups were distributed in different quadrants, suggesting a significant difference in serum metabolic patterns between the CON and HMB groups.

**Figure 3:**
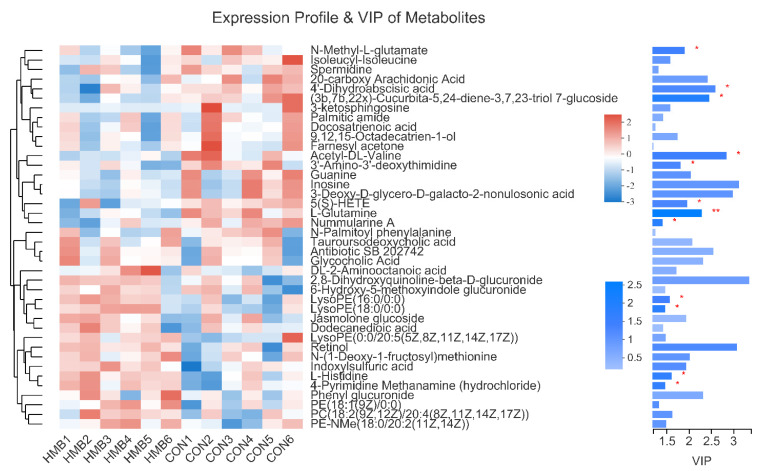
Expression profile and variable importance in the projection (VIP) values of serum metabolites in the control and HMB1 (0.13%) diets. On the left side is the dendrogram of metabolites clustering, the closer the branches are, the closer the expression patterns of all metabolites in the sample are; each column represents a sample, and the bottom is the sample name; each row represents a metabolite, and the color represents the relative expression amount of the metabolite in the group of samples, and the corresponding relationship between the color gradient and the numerical value is shown in the gradient color block. On the right side is the VIP bar graph of metabolites (VIP > 1). The length of the bar represents the contribution value of the metabolite to the difference between the two groups, which is not less than 1 by default. The larger the value, the greater the difference between the two groups. The bar color indicates the significant difference of metabolites between the two groups. Levels of significance are defined as * *p* < 0.05 and ** *p* < 0.01.

Then, a heat map was plotted for the CON vs. HMB (0.13%) chemical compositions to show the alterations in the metabolite concentrations. As shown in Figure 3, the HMB group exhibited higher content of L-histidine and LysoPE(16:0/0:0), and a lower content of *N*-Methyl-l-glutamate and nummularine A (VIP > 1, *p* < 0.05).

### 3.6. The Relationship between Serum Metabolites and Fatty Acid Composition of Muscles

**Figure 4:**
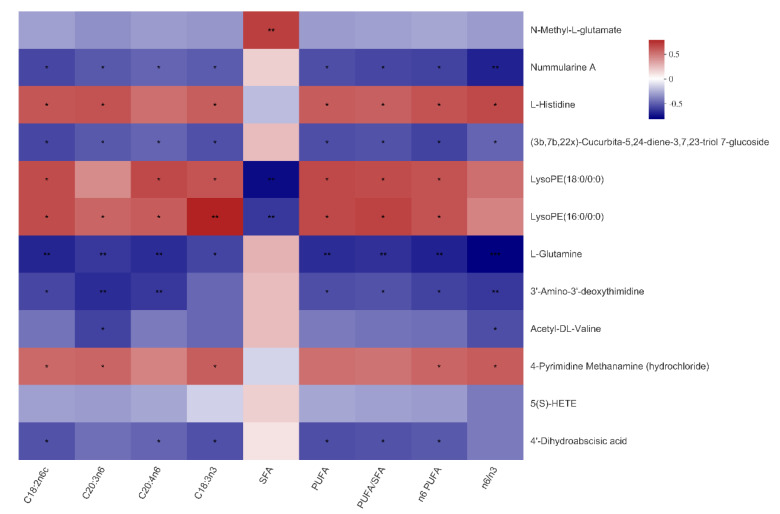
Correlations between serum metabolites and fatty acid composition in the *Longissimus thoracis muscle* of the Bama Xiang mini-pigs. The top 50 metabolites were selected. Levels of significance are defined as * *p* < 0.05, ** *p* < 0.01, and *** *p* < 0.001.

**Figure 5:**
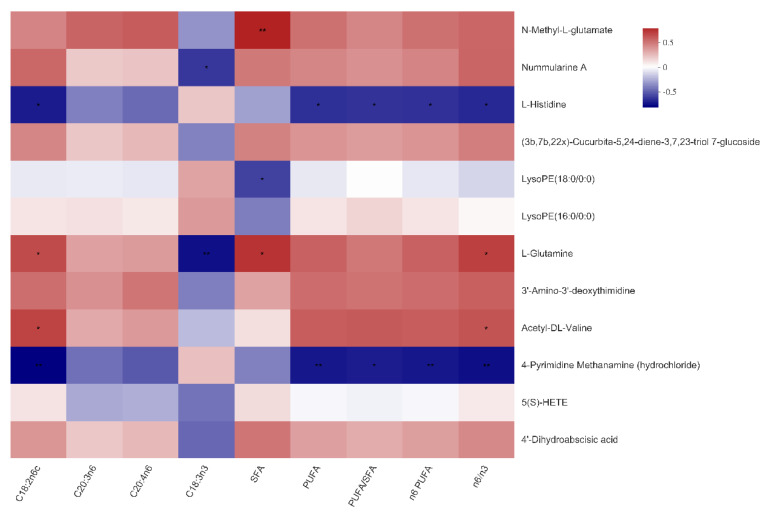
Correlations between serum metabolites and fatty acid composition in the *soleus* muscle of the Bama Xiang mini-pigs. The top 50 metabolites were selected. Levels of significance are defined as * *p* < 0.05 and ** *p* < 0.01.

As shown in Figure 4 and Figure 5, *N*-Methyl-l-glutamate was positively associated with the total SFA content of both LTM and SM (*p* < 0.01), as well as the n6:n3 ratio in the SM (*p* < 0.05). Nummularine A was negatively associated with the C18:3n3 content in the LTM and SM (*p* < 0.05). Of note, the reasons why the number of serum metabolites in Figure 4 and Figure 5 are different from that in Figure 3 are that metabolites in Figure 4 are selected under the condition of VIP ≥ 1.

## 4. Discussion

In response to increasing consumer demands, a major objective of Bama Xiang mini-pig production is to produce minimal visible fat in skeletal muscle without affecting meat quality. In this study, IMF content in LTM was decreased in the pigs fed diets supplemented with 0.13% HMB relative to the CON (3.02% vs. 3.73%), with higher levels of HMB being ineffective. Consistently, our and other’s previous studies have shown that higher HMB concentrations fail to exert beneficial effects on lipid metabolism in adipose tissues of high-fat diet-fed mice [17] and on protein metabolism in skeletal muscles of neonatal pigs [29] and LPS-challenged pigs [30]. Therefore, the results shown in previous studies and in this study suggest that lower levels of HMB are superior to higher levels of HMB in regulating lipid and protein metabolism. However, up to now, the reasons why that happens are still elusive so that it needs to be further explored. In addition, our results indicated that no observable difference in IMF content in SM was noted following HMB treatment. These observations suggest that HMB regulated muscle lipid metabolism in a tissue-specific manner, and the success of increasing fat loss occurred mainly in LTM (muscles mainly composed of fast-twitch glycolytic fibers) rather than in SM (muscles containing predominately slow-twitch oxidative fibers). Muscles with different fiber types possess different morphological, biochemical, and physiological properties. In particular, slow-twitch fibers have more mitochondria than fast-twitch fibers, and fast-twitch fibers grow faster than slow-twitch fibers [31]. Previous studies using growing pigs (Large White × Landrace) have demonstrated that HMB supplementation led to increased mitochondrial biogenesis and fatty acid oxidation in LTM [14]. Therefore, HMB may promote the fat loss of LTM to ensure that there is enough energy to promote its rapid growth. In addition, IMF content is closely related to the characteristics of meat quality, such as juiciness and tenderness. In general, pork with IMF content 2.5–3% exhibits good meat quality and high economic benefits [6,8]. Therefore, HMB could positively affect the meat quality of muscle mainly containing fast-twitch glycolytic fibers, and the appropriate dosage was 0.13%.

Due to its roles in the oxidative stability and nutritional value of muscles, the muscular fatty acid composition is also closely related to meat quality [6,32]. In particular, SFA and PUFA are positively and negatively correlated with meat quality, respectively [5]. However, excessive SFA can affect the degree of fat firmness, subsequently influencing the quality and acceptability of meat products [33]. Human studies have also shown that decreasing dietary SFA and increasing the PUFA:SFA ratio led to a lower risk of cardiovascular diseases [10]. The target for all PUFA:SFA ratio is at least 0.40 [6]. In the current study, although the ratio of all PUFAs to SFAs in selected muscle tissues did not achieve the goal of 0.40 or above, dietary HMB supplementation significantly increased the ratio in LTM relative to the CON, and the maximum elevation of the PUFA:SFA ratio occurred at the HMB level of 0.13% (0.29 vs. 0.10). Similar to IMF content, the PUFA:SFA ratio in SM upon the HMB diets did not achieve the statistical significance in this study. In agreement with these findings, previous studies using growing pigs (Large White × Landrace) also show that HMB supplementation improved fatty acid composition in LTM rather than in SM, as evidenced by increased PUFAs and decreased SFAs as well as SFA/PUFA [34]. However, there is hardly any information available on HMB-affected alterations in muscular fatty acid composition in other animal models, and we do not have a plausible explanation for these observations. Therefore, further investigations are certainly warranted. Apart from the PUFA:SFA ratio, the indices h/H ratio, AI, and TI also can evaluate the effect of fatty acid composition on cardiovascular diseases. Of note, the h/H ratio is more accurate than the PUFA:SFA ratio in reflecting the effect of fatty acid composition on cardiovascular diseases. A relatively high h/H ratio and low content of AI and TI result in a lower incidence of cardiovascular diseases [35]. Consistent with our data of PUFA:SFA ratio, diets supplemented with 0.13% HMB significantly increased the h/H ratio, and decreased AI and TI in the LTM of Bama Xiang mini-pigs, suggesting a beneficial role of HMB in the nutritional value of LTM. Altogether, our results suggested that the synthetic capacity of PUFA in muscles mainly containing fast-twitch glycolytic fibers is improved when Bama Xiang mini-pigs were fed diets supplemented with HMB.

In muscle tissues, the ratio of n6 to n3 PUFAs is mainly regulated by diets since it is difficult to alter this ratio genetically in the short term [36]. Increasing n3 PUFAs and decreasing n6 PUFAs are important to ensure superior meat quality and to increase protection against chronic diseases [37,38]. Therefore, to supply pork rich in valuable n3 PUFAs for humans and improve the health of humans, it is of great importance to increase the proportion of n3 PUFAs and to maintain an appropriate ratio of n6 to n3 PUFAs [2,3,26,39]. The recommended dietary ratio of n6/n3 PUFAs for health benefits is 1:1–2:1 [40], yet the typical Western diet often contains 10 or more times the amount of n6 relative to n3 PUFA [41]. Previous studies using growing pigs (Large White × Landrace) have demonstrated that HMB supplementation could augment n3 PUFAs concentrations in both glycolytic and oxidative skeletal muscles [34]. Similar to the observations made by Zhong et al. [34], we also report here that both LTM and SM of Bama Xiang mini-pigs fed diets supplemented with 0.13% HMB exhibited the greatest increase in the concentrations of n3 PUFAs. However, in parallel, we observed that the n6 PUFAs concentrations and the ratio of n6:n3 PUFAs were also greatly increased in LTM of Bama Xiang mini-pigs. Therefore, our results confirm and extend the observations of Zhong et al. by demonstrating that although not influencing the total PUFA amount, HMB supplementation improved the nutritional value of muscles via enhancing n3 PUFA amount. However, the reasons why HMB differently regulated concentrations of n6 PUFAs in muscle with different fiber types are still elusive, and future studies can pay more attention to the relationship between HMB supplementation and muscular n6 PUFAs metabolism.

The sources of muscular fatty acids are predominately from diets (such as PUFA) and/or lipogenesis (such as SFA), and the destination of muscular fatty acids are mainly mitochondrial oxidation and/or esterification into triglyceride [42]. In this regard, alterations in the muscular fatty acid profile can, to some extent, reflect the balance among fatty acid uptake from food, lipogenesis, and lipolysis. ACC and FAS are two key enzymes for the biosynthesis of long-chain fatty acids (LCFAs) [43]. In the process of lipogenesis, fatty acid transporters such as *FATP1* and *FABP4* facilitate and regulate the entry of exogenous fatty acids into the skeletal muscles [44]. When lipolysis occurs, *HSL* is the key enzyme involved in hydrolyzing stored triglycerides in tissues [45], and *CPT1B* is the key enzyme responsible for transporting LCFAs into the mitochondria for oxidation [43]. Moreover, the expression of lipogenic- and lipolysis-related genes is under the control of nuclear transcription factors such as *C/EBPα* and *PPARγ* [46]. In the current study, 0.13% HMB supplementation affected markers of lipogenesis and lipolysis in LTM of Bama Xiang mini-pigs, as manifested by downregulated mRNA expression of *ACC* and *FAS* and upregulated mRNA expression of *HSL* and *CPT1B*, thus decreasing lipid stores. However, similar observations were not noted in SM. These findings, to some extent, support our hypothesis mentioned above, that is, increased fatty acid oxidation might be a contributing mechanism for HMB-induced fat loss in LTM of Bama Xiang mini-pigs. Taken together, we summarize that an appropriate HMB level could improve lipid metabolism, especially in muscles mainly containing fast-twitch glycolytic fibers, thus regulating IMF content close to the recommended range (2.5–3%).

To further reveal the metabolites and pathways behind the HMB diet regulating the IMF content and fatty acid composition of selected muscles in Bama Xiang mini-pigs, serum samples from the CON and 0.13% HMB diets were subjected to UHPLC-QE-HFX/MS analyses. The total ion chromatogram of the QC samples in positive and negative ion modes supported the stability and reliability of the metabolomics profiles obtained. Moreover, we found that in both LTM and SM of Bama Xiang mini-pigs, *N*-Methyl-l-glutamate was positively correlated with SFA and nummularine A was negatively correlated with C18:3n3 PUFA. In response to HMB supplementation, the two serum metabolites *N*-Methyl-l-glutamate and nummularine A were significantly decreased. Therefore, based on these data and the abovementioned data concerning fatty acid composition, it is postulated that *N*-Methyl-l-glutamate and nummularine A might be potential biomarkers of the HMB-supplemented group and might be implicated in various pathways for lipid metabolism upon HMB supplementation.

In conclusion, the current study indicated that dietary HMB supplementation at the level of 0.13% could improve the IMF content in LTM and fatty acid composition in SM of Bama Xiang mini-pigs. This might be due to the alterations in mRNA expression levels of lipogenic- and lipolysis-related genes in muscle tissues. Additionally, dietary inclusion of HMB regulated the related metabolites (including *N*-Methyl-l-glutamate, L-histidine, and nummularine A) in the serum of pigs. This finding opens a new avenue to understand the mechanisms of action of HMB in improving the meat quality of local pig breeds in China.

## Figures and Tables

**Table 1:** Composition and nutrient levels of the diets (air-dry basis, %) ^a^.

Item	Dietary Levels of HMB, %			
	0	0.13	0.64	1.28
Ingredients, %				
Corn	61.00	62.00	61.21	60.45
Soybean meal	22.00	22.05	22.15	22.60
Wheat bran	14.00	12.82	13.00	12.72
HMB-Ca	0.00	0.13	0.64	1.28
L-Lysine HCl	0.10	0.10	0.10	0.10
Dicalcium phosphate	0.70	0.70	0.70	0.70
Limestone	0.90	0.90	0.90	0.85
Salt	0.30	0.30	0.30	0.30
Premix ^b^	1.00	1.00	1.00	1.00
Total	100.00	100.00	100.00	100.00
Chemical composition, % ^c^				
Digestibe energy, MJ/kg	13.60	13.63	13.55	13.49
Metabolizable energy, MJ/kg	12.15	12.18	12.10	12.03
Dry matter	87.22	87.30	87.35	87.40
Crude protein	15.98	15.94	15.95	16.03
Crude fiber	3.18	3.13	3.16	3.15
Ether extract	3.15	3.13	3.14	3.10
Crude ash	5.96	6.47	6.62	7.06
Calcium	0.61	0.62	0.69	0.76
Total phosphorus	0.54	0.54	0.54	0.54

^a^ Basal diet formulated according to the Chinese National Feeding Standard for Swine. ^b^ Supplied per kg of diet: CuSO4·5H2O 19.8 mg; KI 0.20 mg; FeSO4·7H2O 400 mg; NaSeO3 0.56 mg; ZnSO4·7H2O 359 mg; MnSO4·H2O 10.2 mg; Vitamin K (menadione) 5 mg; Vitamin B1 2 mg; Vitamin B2 15 mg; Vitamin B12 30 μg; Vitamin A 5400 IU; Vitamin D3 110 IU; Vitamin E 18 IU; Choline chloride 80 mg; Antioxidants 20 mg; Fungicide 100 mg. ^c^ Digestible energy, metabolizable energy, calcium, and total phosphorus are calculated values, and other reported data are measured values.

**Table 2:** Amino acid concentrations in the serum of Bama Xiang mini-pigs fed diets with different levels of beta-hydroxy beta-methyl butyrate (HMB) (*n* = 8).

Amino Acid (AA, nmol/mL) ^1^	Dietary Levels of HMB, %				*p*-Value
	0	0.13	0.64	1.28	
Nutritionally EAA					
L-leucine	30.41 ± 2.38	33.33 ± 2.04	30.52 ± 1.79	28.05 ± 1.10	0.299
L-isoleucine	16.73 ± 1.52	20.73 ± 2.45	18.29 ± 2.06	16.03 ± 0.72	0.294
L-valine	41.98 ± 3.15	47.09 ± 3.28	43.37 ± 3.21	41.13 ± 2.23	0.525
L-histidine	9.60 ± 0.44 ^b^	11.65 ± 0.62 ^a,b^	12.37 ± 0.61 ^a^	11.60 ± 0.70 ^a,b^	0.023
L-lysine	19.29 ± 1.47	23.66 ± 2.17	19.51 ± 0.25	19.82 ± 1.53	0.170
L-methionine	2.16 ± 0.11 ^ab^	2.54 ± 0.16 ^a^	2.02 ± 0.06 ^b^	1.98 ± 0.11 ^b^	0.009
L-phenylalanine	18.07 ± 1.22	18.39 ± 0.69	18.99 ± 0.58	17.61 ± 0.94	0.740
L-threonine	12.52 ± 0.84 ^ab^	14.61 ± 1.22 ^a^	9.82 ± 0.70 ^b^	12.84 ± 1.85 ^a,b^	0.084
Total EAA	150.76 ± 9.06	171.99 ± 9.23	154.89 ± 8.05	149.07 ± 4.79	0.193
Nutritionally NEAA					
L-alanine	24.88 ± 0.72 ^b^	41.71 ± 3.30 ^a^	35.28 ± 4.21 ^a,b^	38.34 ± 4.04 ^a^	0.013
L-arginine	30.21 ± 1.20 ^b^	33.46 ± 0.31 ^a,b^	30.65 ± 1.31 ^a,b^	34.58 ± 0.87 ^a^	0.014
L-aspartic acid	1.75 ± 0.28 ^a^	0.97 ± 0.09 ^b^	1.43 ± 0.23 ^a,b^	1.80 ± 0.16 ^a^	0.034
L-glutamic acid	51.57 ± 3.56	59.49 ± 3.63	55.40 ± 2.15	57.34 ± 6.35	0.599
L-glycine	39.28 ± 3.42	38.86 ± 0.86	30.40 ± 2.94	39.37 ± 3.46	0.105
L-serine	10.68 ± 0.92	12.15 ± 0.57	10.63 ± 0.71	11.36 ± 0.22	0.348
L-tyrosine	10.10 ± 0.67 ^a,b^	11.52 ± 0.33 ^a^	8.63 ± 0.53 ^b^	9.78 ± 0.74 ^a,b^	0.020
L-proline	16.80 ± 0.53	17.63 ± 1.13	19.05 ± 1.97	17.46 ± 1.59	0.721
Total NEAA	185.27 ± 9.12	215.79 ± 6.59	191.47 ± 11.34	210.03 ± 9.04	0.087
Other AA					
*O*-phosphoserine	1.04 ± 0.10 ^b^	1.31 ± 0.06 ^a^	0.84 ± 0.04 ^b,c^	0.65 ± 0.04 ^c^	<0.001
Taurine	30.43 ± 1.86	31.28 ± 2.09	33.55 ± 2.09	29.37 ± 2.77	0.602
L-α-amino-adipic acid	10.67 ± 1.44	11.55 ± 1.11	8.83 ± 0.47	8.68 ± 0.24	0.119
L-citrulline	4.82 ± 0.46	4.58 ± 0.20	4.22 ± 0.48	4.28 ± 0.51	0.742
L-α-amino-n-butyric acid	0.74 ± 0.12	0.90 ± 0.16	0.90 ± 0.14	1.25 ± 0.15	0.113
L-cystathionine	1.93 ± 0.27	1.40 ± 0.05	2.03 ± 0.24	2.18 ± 0.41	0.247
β-alanine	0.75 ± 0.01 ^a^	0.32 ± 0.07 ^b^	0.88 ± 0.12 ^a^	0.26 ± 0.04 ^b^	<0.001
L-ornithine	6.25 ± 0.46 ^b^	8.74 ± 0.61 ^a^	6.17 ± 0.43 ^b^	7.70 ± 0.86 ^a,b^	0.022
3-methyl-l-histidine	2.35 ± 0.21	2.32 ± 0.20	2.74 ± 0.36	2.72 ± 0.14	0.458

^1^ EAA: essential amino acid; NEAA: non-essential amino acid. ^a,b,c^ Values within a row with different superscripts differ significantly (*p* < 0.05).

**Table 3:** Serum lipid-related substances of Bama Xiang mini-pigs fed the diets with various levels of HMB (*n* = 8).

Items ^1^	Dietary Levels of HMB, %				*p*-Values
	0	0.13	0.64	1.28	
G6PD, ng/mL	9.28 ± 0.22 ^a^	5.76 ± 0.30 ^b^	9.53 ± 0.18 ^a^	6.71 ± 0.33 ^b^	<0.001
ATGL, ng/mL	388.12 ± 24.41 ^a,b^	428.76 ± 21.07 ^a^	251.42 ± 19.16 ^c^	315.70 ± 12.07 ^b,c^	<0.001
LPL, ng/mL	5.21 ± 0.44 ^b^	2.68 ± 0.45 ^c^	7.13 ± 0.39 ^a^	3.52 ± 0.41 ^c^	<0.001
HMGR, ng/mL	58.77 ± 2.98 ^b^	64.81 ± 4.57 ^b^	89.54 ± 6.06 ^a^	76.18 ± 7.39 ^a,b^	0.004

^1^ G6PD: glucose-6-phosphate dehydrogenase; ATGL: adipose tissue triglyceride lipase; LPL: lipoprotein lipase; HMGR: 3-hydroxy-3-methylglutaryl coenzyme A reductase. ^a,b,c^ Values within a row with different superscripts differ significantly (*p* < 0.05).

**Table 4:** Fatty acid composition of *Longsissimus thoracis muscle* in Bama Xiang mini-pigs fed the diets with various levels of HMB (% of total fatty acids) (*n* = 8).

Items	Dietary Levels of HMB, %				*p*-Value
	0	0.13	0.64	1.28	
C14:0	2.07 ± 0.12 ^a^	1.43 ± 0.05 ^b^	1.88 ± 0.15 ^a^	1.83 ± 0.13 ^a^	0.008
C16:0	31.86 ± 0.44 ^a^	28.53 ± 0.37 ^b^	30.06 ± 0.53 ^a,b^	30.42 ± 0.79 ^a,b^	0.004
C16:1	2.77 ± 0.14	2.05 ± 0.20	2.67 ± 0.22	2.64 ± 0.21	0.068
C17:0	0.21 ± 0.01	0.20 ± 0.01	0.18 ± 0.01	0.18 ± 0.01	0.025
C18:0	17.86 ± 0.53	18.50 ± 0.52	17.34 ± 0.27	17.83 ± 0.16	0.267
C18:1 n9t	0.19 ± 0.01	0.18 ± 0.03	0.14 ± 0.01	0.17 ± 0.01	0.255
C18:1 n9c	37.63 ± 0.42	32.89 ± 2.41	35.44 ± 0.60	35.21 ± 1.13	0.150
C18:2 n6c	4.22 ± 0.51 ^b^	10.14 ± 1.19 ^a^	7.50 ± 0.57 ^a,b^	7.18 ± 1.08 ^a,b^	0.002
C20:0	0.25 ± 0.01	0.25 ± 0.02	0.28 ± 0.01	0.23 ± 0.01	0.176
C20:1	1.53 ± 0.11	1.19 ± 0.09	1.32 ± 0.08	1.21 ± 0.05	0.044
C18:3 n3	0.20 ± 0.02 ^b^	0.26 ± 0.01 ^a^	0.25 ± 0.01 ^a,b^	0.23 ± 0.02 ^a,b^	0.033
C20:2	0.19 ± 0.03 ^b^	0.37 ± 0.05 ^a^	0.32 ± 0.01 ^a,b^	0.29 ± 0.04 ^a,b^	0.010
C20:3 n6	0.14 ± 0.01 ^b^	0.40 ± 0.06 ^a^	0.25 ± 0.03 ^a,b^	0.24 ± 0.05 ^a,b^	0.005
C20:4 n6	0.66 ± 0.09 ^b^	3.26 ± 0.74 ^a^	1.97 ± 0.27 ^a,b^	1.80 ± 0.46 ^a,b^	0.007
SFA ^1^	52.25 ± 0.57 ^a^	48.92 ± 0.80 ^b^	49.73 ± 0.60 ^a,b^	50.48 ± 0.80 ^a,b^	0.020
PUFA ^2^	5.41 ± 0.64 ^b^	14.43 ± 1.86 ^a^	10.29 ± 0.85 ^a,b^	9.73 ± 1.64 ^a,b^	0.002
∑PUFA:SFA	0.10 ± 0.01 ^b^	0.29 ± 0.03 ^a^	0.21 ± 0.02 ^a,b^	0.19 ± 0.03 ^a,b^	0.001
∑n3 PUFA ^3^	0.20 ± 0.02 ^b^	0.26 ± 0.01 ^a^	0.25 ± 0.01 ^a,b^	0.23 ± 0.02 ^a,b^	0.033
∑n6 PUFA ^4^	5.02 ± 0.60 ^b^	13.80 ± 1.83 ^a^	9.73 ± 0.85 ^a,b^	9.22 ± 1.58 ^a,b^	0.002
∑n6:n3 PUFA	24.88 ± 2.10 ^b^	52.83 ± 6.95 ^a^	39.68 ± 3.49 ^a,b^	38.82 ± 4.21 ^a,b^	0.004
h/H ^5^	1.27 ± 0.03 ^b^	1.58 ± 0.05 ^a^	1.43 ± 0.05 ^a,b^	1.40 ± 0.07 ^a,b^	0.005
AI ^6^	0.84 ± 0.02 ^a^	0.67 ± 0.02 ^b^	0.75 ± 0.03 ^a,b^	0.77 ± 0.04 ^a,b^	0.004
TI ^7^	2.13 ± 0.05 ^a^	1.87 ± 0.06 ^b^	1.93 ± 0.05 ^a,b^	1.99 ± 0.07 ^a,b^	0.019

^1^ SFA = C14:0 + C16:0 + C17:0 + C18:0 + C20:0, ^2^ PUFA = C18:2n6c + C18:3n3 + C20:2+ C20:3n6 + C20:4n6, ^3^ n3 PUFA = C18:3n3, ^4^ n6 PUFA = C18:2n6c + C20:3n6 + C20:4n6, ^5^ h/H (Hypocholesterolemic/Hypercholesterolemic ratio) = (C18:1n9t + C18:1n9c + C18:2n6c + C18:3n3 + C20:3n6 + C20:4n6)/(C14:0 + C16:0), ^6^ AI (atherogenicity index) = (4*C14:0 + C16:0)/(100 − SFA), ^7^ TI (thrombogenicity index) = (C14:0 + C16:0 + C18:0)/(0.5*MUFA + 0.5*∑n6 PUFA + 3*C18:3n3 + 1/(∑n6:n3 PUFA)). ^a,b,^ Values within a row with different superscripts differ significantly (*p* < 0.05).

**Table 5:** Fatty acid composition of *Soleus* muscle in Bama Xiang mini-pigs fed the diets with various levels of HMB (% of total fatty acids) (*n* = 8).

Items	Dietary Levels of HMB, %				*p*-Value
	0	0.13	0.64	1.28	
C14:0	1.35 ± 0.12	1.28 ± 0.15	1.48 ± 0.12	1.37 ± 0.09	0.711
C16:0	28.16 ± 0.56	27.92 ± 0.62	29.07 ± 0.49	29.32 ± 0.38	0.197
C16:1	1.48 ± 0.21	1.98 ± 0.29	1.80 ± 0.20	1.74 ± 0.12	0.418
C17:0	0.27 ± 0.03	0.24 ± 0.02	0.21 ± 0.01	0.20 ± 0.01	0.095
C18:0	20.08 ± 0.88	17.93 ± 0.56	19.73 ± 0.62	20.28 ± 0.39	0.062
C18:1 n9t	0.28 ± 0.05	0.19 ± 0.02	0.20 ± 0.02	0.21 ± 0.02	0.224
C18:1 n9c	25.09 ± 2.08	31.66 ± 3.45	29.77 ± 2.07	28.81 ± 1.90	0.313
C18:2 n6c	15.21 ± 1.51 ^a^	11.04 ± 1.05 ^b^	11.28 ± 1.30 ^b^	11.45 ± 0.83 ^b^	0.072
C20:0	0.24 ± 0.02 ^a,b^	0.22 ± 0.01 ^b^	0.27 ± 0.01 ^a^	0.24 ± 0.01 ^a,b^	0.058
C20:1	0.94 ± 0.12	0.86 ± 0.07	1.08 ± 0.12	0.94 ± 0.06	0.450
C18:3 n3	0.27 ± 0.00 ^a,b^	0.29 ± 0.01 ^a^	0.26 ± 0.00 ^a,b^	0.25 ± 0.01 ^b^	0.014
C20:2	0.40 ± 0.02	0.34 ± 0.02	0.37 ± 0.02	0.37 ± 0.02	0.243
C20:3 n6	0.54 ± 0.06	0.41 ± 0.10	0.42 ± 0.07	0.43 ± 0.06	0.571
C20:4 n6	5.11 ± 0.50	3.62 ± 0.95	3.60 ± 0.55	3.90 ± 0.66	0.380
SFA ^1^	50.09 ± 0.66 ^a^	47.58 ± 0.70 ^b^	50.75 ± 0.56 ^a^	51.41 ± 0.65 ^a^	0.003
PUFA ^2^	21.53 ± 2.06	17.37 ± 3.46	15.92 ± 1.92	16.40 ± 1.48	0.343
∑PUFA:SFA	0.43 ± 0.04	0.36 ± 0.07	0.31 ± 0.04	0.32 ± 0.03	0.292
∑n3 PUFA ^3^	0.27 ± 0.00 ^a,b^	0.29 ± 0.01 ^a^	0.26 ± 0.00 ^ab^	0.25 ± 0.01 ^b^	0.014
∑n6 PUFA ^4^	20.86 ± 2.04	15.07 ± 2.03	15.30 ± 1.92	15.78 ± 1.48	0.014
∑n6:n3 PUFA	78.09 ± 7.10 ^a^	52.33 ± 6.54 ^b^	59.26 ± 7.41 ^a,b^	63.81 ± 7.06 ^a,b^	0.099
h/H ^5^	1.58 ± 0.05	1.62 ± 0.07	1.49 ± 0.04	1.47 ± 0.04	0.173
AI ^6^	0.67 ± 0.02	0.63 ± 0.03	0.71 ± 0.02	0.72 ± 0.02	0.064
TI ^7^	1.95 ± 0.05 ^a^	1.76 ± 0.05 ^b^	2.01 ± 0.04 ^a^	2.06 ± 0.05 ^a^	0.002

^1^ SFA = C14:0 + C16:0 + C17:0 + C18:0 + C20:0. ^2^ PUFA = C18:2n6c + C18:3n3 + C20:2+ C20:3n6 + C20:4n6, ^3^ n3 PUFA = C18:3n3, ^4^ n6 PUFA = C18:2n6c + C20:3n6 + C20:4n6, ^5^ h/H (Hypocholesterolemic/Hypercholesterolemic ratio) = (C18:1n9t + C18:1n9c + C18:2n6c + C18:3n3 + C20:3n6 + C20:4n6)/(C14:0 + C16:0), ^6^ AI (atherogenicity index) = (4*C14:0 + C16:0)/(100 − SFA), ^7^ TI (thrombogenicity index) = (C14:0 + C16:0 + C18:0)/(0.5*MUFA + 0.5*∑n6 PUFA + 3*C18:3n3 + 1/(∑n6:n3 PUFA)). ^a,b,^Values within a row with different superscripts differ significantly (*p* < 0.05).

## Data Availability

The data presented in this study are available on request from the corresponding author.

## Supplementary Materials

The following are available online at https://www.mdpi.com/article/10.3390/ani11051190/s1, Table S1: Primers used for real-time PCR; Table S2: Growth performance and intramuscular fat of Bama Xiang mini-pigs fed the diets with various levels of HMB (*n* = 8); Figure S1: The total ion chromatogram of serum QC samples in positive (A) and negative (B) ion modes based on UHPLC-QE-HFX/MS platform.
