# Supplementary material for: Alterations of the Muscular Fatty Acid Composition and Serum Metabolome in Bama Xiang Mini-Pigs Exposed to Dietary Beta-Hydroxy Beta-Methyl Butyrate
Source: Animals (Basel). 2021 Apr 21;11(5):1190. doi: 10.3390/ani11051190 (PMC8143165; doi:10.3390/ani11051190)
Supplement: Supplementary file 1 [file animals-11-01190-s001.zip › animals-1170980-supplementary.pdf]

Table S1. Primers used for real-time PCR.

Genes.	Primers	Sequences (5′-3′)	Size (bp)	Accession No.	T_A (°C)[1]
ACC	Forward	ATCCCTCCTTGCCTCTCCTA	208	NM_001114269	58
	Reverse	ACTTCCCGTTCAGATTTCCG			
FAS	Forward	CTACCTTGTGGATCACTGCATAGA	114	NM_001099930	60
	Reverse	GGCGTCTCCTCCAAGTTCTG			
HSL	Forward	GCAGCATCTTCTTCCGCACA	195	HM_591297	62
	Reverse	AGCCCTTGCGTAGAGTGACA			
CPT1B	Reverse	ACTGTCTGGGCAAACCAAAC	176	NM_001007191	60
	Forward	CTTCTTGATGAGGCCTTTGC			
FATP-1	Reverse	GGAGTAGAGGGCAAAGCAGG	208	JN713898	64
	Forward	AGGTCTGGCGTGGGTCAAAG			
FABP4	Reverse	TGGAAACTTGTCTCCAGTG	147	HM_453202	58
	Forward	GGTACTTTCTGATCTAATGGTG			
C/EBPα	Reverse	AACAACTGAGCCGCGAACTGGA	170	XM_003127015	60
	Forward	CTTGAGATCTGGAGACCCGAAACC			
PPARγ	Reverse	TGACCATGGTTGACACCG	381	XM_005669790	58
	Forward	AAGCATGAACTCCATAGTGG			
β-actin	Forward	TGCGGGACATCAAGGAGAAG	216	XM_003357928.2	64
	Reverse	AGTTGAAGGTGGTCTCGTGG			

[1] T_A: annealing temperature.

Table S2. Growth performance and intramuscular fat of Bama Xiang mini-pigs fed the diets with various levels of HMB (n = 8)[1].

Items[2]	Dietary levels of HMB, %				SEM	*P*-values
	**0**	0.13	0.64	1.28		
Initial BW, kg	8.62	8.56	8.58	8.57	0.403	0.999
Final BW, kg	24.73^{b}	26.87^{a}	24.84^{b}	25.37^{b}	0.422	0.021
ADG, g/d	268.54^{b}	305.10^{a}	271.04^{b}	279.98^{ab}	1.808	0.036
ADFI, g/d	1085.90	1195.13	1093.00	1137.79	3.825	0.241
F:G	4.05	3.91	4.06	4.09	0.224	0.817
Intramuscular fat (%)						
Longsissimus thoracis muscle	3.73^{a}	3.02^{b}	3.61^{a}	3.63^{a}	0.235	0.002
Soleus muscle	3.66	3.30	3.60	3.53	0.226	0.171

[1] The data about growth performance has been published and can be found in the following reference, that is, Zheng CB, Song B, Zheng J, et al. Effects of dietary supplementation of beta-hydroxy-beta-methylbutyrate on growth performance and liver lipid metabolism of Bama Xiang pigs. *Chinese Journal of Animal Nutrition*, 2021, 33(7): https://kns.cnki.net/kcms/detail/11.5461.S.20210316.0920.012.html. [2] ADG, average daily gain; ADFI, average daily feed intake; F:G, feed:gain. [a,b] Means with different superscripts within a row differ (P < 0.05).

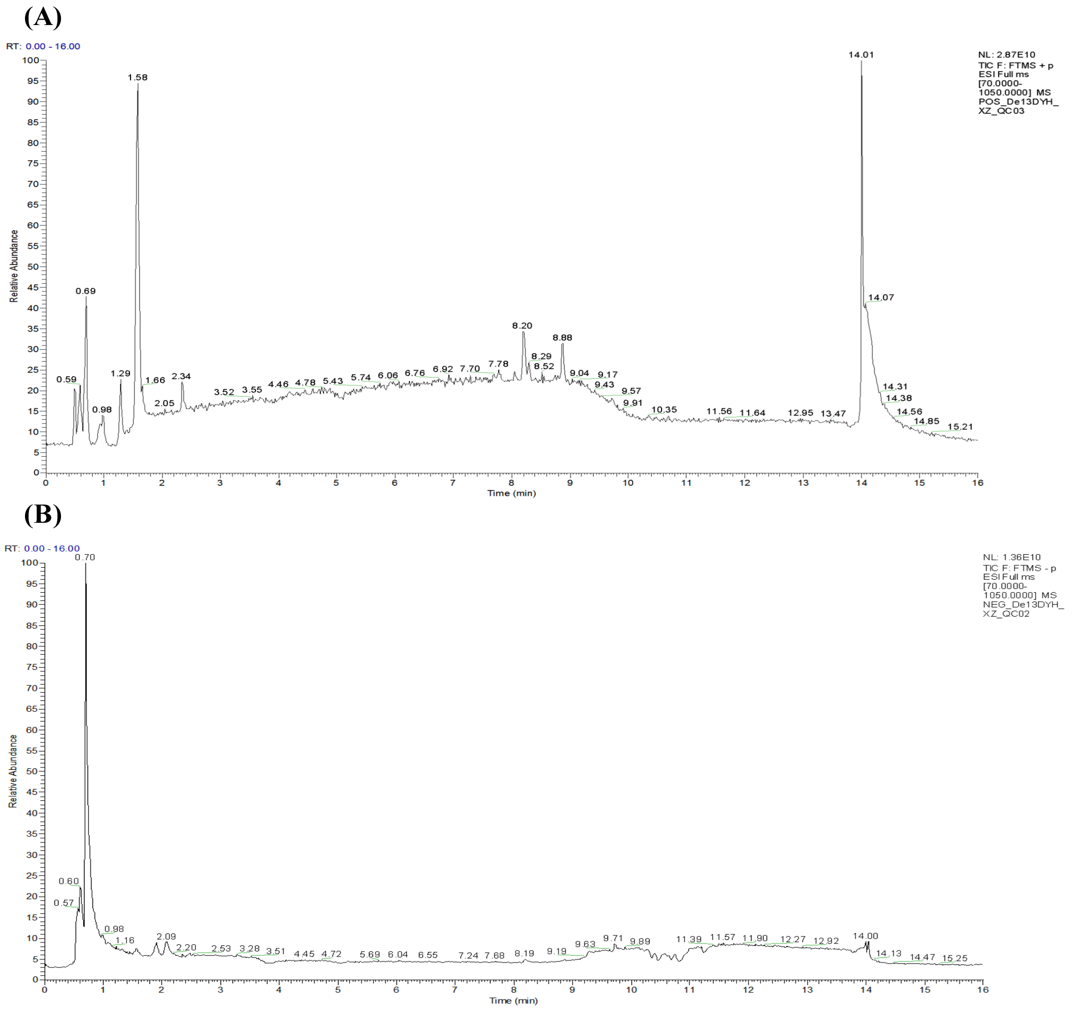

Figure S1. The total ion chromatogram of serum QC samples in positive (A) and negative (B) ion modes based on UHPLC-QE-HFX/MS platform.
